# Application and comparison of five risk assessment methods in ferrous metal foundry enterprises with silica dust concentration exceeding the limit posts

**DOI:** 10.3389/fpubh.2025.1465284

**Published:** 2025-02-06

**Authors:** Qimeng Li, Xiaoying Li, Ge Qin, Yuanmeng Qi, Jie Liu, Xiaocui Tang, Di Wu, Changfu Hao, Sihua Wang

**Affiliations:** ^1^Department of Occupational Health, College of Public Health, Zhengzhou University, Zhengzhou, China; ^2^Department of Surgery, The Third Affiliated Hospital of Zhengzhou University, Zhengzhou, China; ^3^Department of Occupational Health, The Third People's Hospital of Henan Province (Henan Hospital for Occupational Diseases), Zhengzhou, China

**Keywords:** silica dust exposure, risk assessment, occupational health, ferrous metal manufacturing, occupational exposure risk, ferrous foundries

## Abstract

**Background:**

Explore methods to accurately reflect the risk level of ferrous metal foundry workplaces when the silica dust concentration exceeds the limit, and provide a basis for the application of risk assessment techniques in key industries with occupational exposure to silica dust.

**Methods:**

The survey was conducted in 25 ferrous metal casting industries in Henan Province, China. Five occupational health risk assessment (OHRA) methods, including Risk index method, Hazard grading method, International Council on Mining and Metals model, The synthesis index method, and The exposure ratio method, were used to assess the occupational health risk of the workplaces that produced silica dust and the concentration of silica dust exceeded the occupational exposure limits (OELs), and to compare the results of the different methods.

**Results:**

The risk index assessment method yielded one job of mild hazard, seven job of moderate hazard, fifteen jobs of high hazard, and forty-four jobs of extreme hazard. The hazard classification method resulted in two jobs of mild hazard, six jobs of moderate hazard, and fifty-nine jobs of high hazard. The ICMM qualitative method identified fifteen jobs of moderate risk and fifty-two jobs of high risk. The synthesis index method revealed nine jobs of moderate risk and fifty-eight jobs of high risk. The exposure ratio method identified ten jobs of high risk and fifty-seven jobs of extremely high risk. The results obtained from the synthesis index method showed relatively lower levels, except for the index method, there was a certain correlation (*r*: 0.541–0.798, *P* < 0.05) and consistency (*kapa*: 0.521–0.561, *P* < 0.05) with the remaining four methods.

**Conclusion:**

This study shows that although there are some differences in the results of different OHRA methods, there is also some correlation between them, which can corroborate each other and enhance the reliability of the assessment results. In practical application, appropriate assessment methods should be selected according to specific situations and the results of multiple methods should be combined and analyzed comprehensively to ensure accurate identification and assessment of occupational hazards and provide a scientific basis for improving occupational safety and health management.

## Introduction

The ferrous metal casting industry refers to enterprises that use ferrous metal materials such as iron and steel, which are melted and injected into casting molds and solidified to form various castings or parts. It is an important branch of the global manufacturing industry, widely used in machinery manufacturing, automotive industry, aerospace and other fields, with a large number of enterprises. This industry should be focused on when carrying out occupational disease prevention and control work. Occupational health risk assessment (OHRA) refers to the process of qualitatively or quantitatively measuring the level of occupational health risks by comprehensively and systematically identifying and analyzing the risk factors and protective measures in the workplace, so as to take appropriate control measures (1–3). It is necessary to carry out occupational risk assessment for the ferrous metal casting industry.

This industry involves high-temperature melting and complex production processes, and workers are often exposed to harmful chemicals and dusts, among which respirable crystalline silica (RCS) is one of the most common (4). RCS is hazardous to the human respiratory system and the atmospheric environment (5). Operators often inhale silica dust at high concentrations for long periods of time, and if inhaled it can result in extensive nodular fibrosis of the lungs, which in severe cases affects lung function (6).

Occupational health risk assessment can systematically identify and analyze potential hazards in the workplace and help companies understand the risk level of different jobs. OHRA not only identifies existing safety hazards, but also provides enterprises with improvement measures, thereby reducing the incidence of accidents and safeguarding the safety of employees. Furthermore, with the development of the industry and the advancement of technology, many small foundries still suffer from insufficient safety management and occupational health awareness, even though enterprises have taken certain protective measures. Through systematic risk assessment, the sources of hazards in the workplace can be identified and analyzed, providing the scientific basis for the formulation of effective control measures.

The assessment results can provide the data supports for the industry regulators to help formulate more scientific and reasonable occupational health policies and standards, and improve the level of safe production in the whole industry. Therefore, carrying out OHRA in the ferrous metal foundry industry not only helps to protect workers' health, but is also a key initiative to promote the stable development of the industry.

The right assessment method can improve the reliability and validity of the results, ensure that the assessment process is in line with industry best practice, thus enhancing the authority of the results, and facilitating communication and implementation both within and outside the organization. In this study, the Occupational hazard risk index assessment method (7), the Hazard grading method (8), the International Council on Mining and Metals (ICMM) OHRA method (referred to as the “ICMM assessment method”) (9), the exposure ratio assessment method (referred to as “The exposure ratio”) outlined in the “Guidelines for occupational health risk assessment of chemicals in the workplace (GBZ/T 298-2017)” (10), and the synthesis index method (11) were used to 25 black metal casting enterprises in Henan Province, which are exposed to silica dust. These assessments aim to verify the applicability of these five methods in assessing the risks associated with silica dust concentration exceeding the limit value of 0.3 mg/m^3^ (12) in the ferrous metal casting industry. The goal is to scientifically guide employers in implementing occupational health management practices and to provide a scientific basis for preventing and controlling silicosis in this industry.

## Materials and methods

### Subjects

The survey respondents of this study were selected from the key positions of 25 ferrous metal casting enterprises (including one large-scale enterprise, one medium-sized enterprise, eight small-scale enterprises, and 15 micro-enterprises) in Henan Province, China, in order to ensure the representativeness of the sample and the diversity of the data. Specifically, the criteria for classifying the size of the enterprises were based on the number of employees: small enterprises had <50 employees, medium-sized enterprises had between 50 and 200 employees, and large enterprises had more than 200 employees. The average number of years workers have worked in the foundry industry is 7 years, with the majority of workers concentrated between 5 and 12 years. Selection criteria for key positions: (1) Silica dust is generated and the concentration of silica dust detected exceeds the occupational exposure limit. (2) Workers in the position have a high frequency of exposure to silica dust. (3) Workers in the position may have inadequate protective measures. (4) As a typical position in the ferrous metal foundry industry (5) Potential for improvement.

### Methods

#### The on-site occupational health investigation

The OHRA questionnaire designed by our research group was used to investigate the basic situation of enterprise. The survey focuses on enterprise fundamentals (labor allocation, production system, etc.), worker exposure conditions (mode of operation, level of exposure, duration of exposure, frequency of exposure, etc.), as well as aspects of health engineering protection, emergency response facilities and measures, personal protective equipment, and occupational health management.

#### Detection of occupational hazard factors

Occupational hazard factor detection involves sampling and testing the levels of silica dust in workplace air, following the specifications outlined in “Specifications of air sampling for hazardous substances monitoring in the workplace (GBZ159-2004)” (13). Short-term exposure concentrations (C-STEL) and time-weighted average concentrations (C-TWA) of silica dust are measured at each workstation. The results are then assessed according to “Occupational exposure limits for hazardous agents in the workplace Part 1: Chemical hazardous agents (GBZ 2.1-2019)” (12).

### HRA methods

#### Occupational hazard risk index method

The methodology was proposed by Sihao et al. (14) and aims to assess the health impact of hazards in the workplace. It takes into account the severity of the hazards, the likelihood of their occurrence, and the conditions of the working environment, and thus establishes a system of indicators for OHRA. Specifically, a formula is used to calculate the occupational hazard risk index. The formula is: “risk index = 2^health effect level^ × 2^exposure ratio^ × operating condition level”. Among them, the “health effect level” of dust was divided into four levels (15). The “exposure ratio” is determined as the ratio of the average measured value to the OEL; and the “operating condition level” is calculated as the fourth root of the product of exposure time level, exposure population level, engineering protection measure level, and individual protection measure level. Based on the magnitude of the risk index, the risk level is classified into five levels: 0–6, no hazard; >6–11, mild hazard; >11–23, moderate hazard; >23–80, high hazard; >80~ +∞, extreme hazard (16).

#### ICMM method (qualitative evaluation)

The ICMM method refers to a risk assessment approach proposed by the International Council on Mining and Metals (9), primarily used to evaluate various risks involved in the mining and metal industries, including environmental, social, health, and safety risks. The methodology is presented in a matrix format, whereby the assessor determines risk ratings for different levels of exposure based on the identified health hazards, combined with (OELs). These levels are categorized as low, medium and high risk. During the assessment, the personnel also make a qualitative assessment by considering the possible health consequences at a given exposure level (17), allowing enterprises to better understand the risk levels and implement corresponding management measures to mitigate the impact of risks.

#### Hazard grading method

The hazard level is calculated based on the formula G = W_M_ × W_B_ × W_L_ as outlined in “Classification of occupational hazards at workplaces. Part 1: Occupational exposure to industrial dust (GBZ/T 229.1-2010)” (8). Here, W_M_ represents the content of free silica in dust, W_B_ denotes the exposure ratio to airborne dust, and W_L_ indicates the labor intensity. G is classified into four levels: relatively harmless operations (G = 0, Grade 0), mild hazard operations (0 < G ≤ 6, Grade I), moderate hazard operations (6 < G ≤ 16, Grade II), and high hazard operations (G > 16, Grade III).

#### Synthesis index method

This method, based on the Singapore method (18), is utilized to determine the magnitude of the risk index R and subsequently establish risk levels (19). The calculation formula for R is R= $\sqrt{HR\times ER}$, where HR is the hazard rating, based on the toxicity of the chemical hazardous factor. For example, the hazard level of silica dust is 5. ER denotes the exposure rating, calculated as ER = [EI_1_xEI_2_……EI_N_]/n. “EI” here is the exposure index, which takes into account a number of factors, including the aerodynamic diameter of the chemically hazardous substance, the ratio of the exposure level to the OELs, occupational disease prevention and control measures, the amount of use and the duration of exposure, and so on. “n” represents the exposure factor of the exposure factor.

#### Exposure ratio method

This method is used when we have access to test results for hazardous substances in the air and the appropriate OELs have been established. The actual measured exposure concentration (E) is compared to the OELs and the maximum value of E/OEL is calculated. This maximum value helps us to determine the risk level of exposure and thus assess the safety of the workplace (10).

### Comparison of risk assessment results

The risk level classifications obtained from the five assessment methods used in this study were not entirely consistent. In order to make the risk levels obtained from different methods comparable, standardized processing of the risk levels obtained from different assessment methods was performed, converting them into risk ratios (RRs) for comparative analysis (20). The formula used was RR = R/N (R = the risk level; N = the highest risk level obtained from the method used). RR was categorized as follows: 0–0.2, level 1; >0.2–0.4, level 2; >0.4–0.6, level 3; >0.6–0.8, level 4; >0.8–1.0, level 5. Since the risk ratios do not follow a normal distribution, Spearman correlation analysis was used to evaluate the correlation between the risk ratios of different methods. There are five levels of consistency: 0– <0.2, indicated poor consistency; 0.2– <0.4, fair correlation; 0.4– <0.6, moderate correlation; 0.6– <0.8, strong correlation; 0.8– <1.0, very strong correlation. SPSS 20.0 software was used to compare the results of various assessment models, and the consistency of risk ratio levels between each pair of assessment methods was examined using Kappa consistency. There are four levels of Kappa consistency: 0– <0.4, poor consistency; 0.4– <0.6, fair consistency; 0.6– <0.8, high consistency; >0.8, very good consistency.

## Results

### On-site investigation results

The on-site investigation and testing data were obtained from the Third People's Hospital of Henan Province, China. There were 25 black metal casting enterprises, all of which, were comprised one large-scale enterprise, one medium-sized enterprise, eight small-scale enterprises, and fifteen micro-enterprises. Their production processes were largely similar, involving pattern design, melting and casting, cooling and demoulding, and surface treatment. There were five positions with exposure to silica dust, including molding sand, melting, casting, cleaning, and grinding, with a total of 256 workers involved. Among these positions, six enterprises exceeded the standard silica dust concentration at one position, four enterprises at two positions, six enterprises at three positions, and nine enterprises at four positions. Except for two enterprises, which operated on a 6-h shift, the remaining 23 enterprises followed an 8-h work schedule. Only one enterprise had well-established dust and poison prevention facilities, as well as comprehensive emergency response measures. They provided adequate dust masks and gas masks to meet the needs of all employees, ensuring that each employee has suitable protective equipment for their work and that implementation is relatively good. However, the dust and poison prevention facilities and emergency response measures in other enterprises were incomplete or ineffective. While most enterprises had established occupational health management systems, their implementation was generally poor.

### Evaluation results using five methods

The risk index method identified one job of low risk, seven jobs of moderate risk, 15 jobs of high risk, and 44 jobs of extreme risk. The hazard grading method yielded two jobs of low risk, six jobs of moderate risk, and 59 jobs of high risk. The ICMM method identified 15 jobs of moderate risk and 52 jobs of high risk. The synthesis index method resulted in nine jobs of moderate risk and 58 jobs of high risk. The exposure ratio method found 10 jobs of high risk and 57 jobs of extremely high risk.

As shown in Table 1, all assessment methods yielded relatively high-risk levels, consistent with on-site measurements indicating silica dust concentrations exceeding occupational exposure limits. Various positions with elevated silica dust concentrations pose a certain risk of health damage.

**Table 1:** Occupational health risk assessment results of silica dust in different positions in different enterprises.

**Enterprise**	**Location**	**Number of workers**	**C_TWA_ (mg/m^3^)**	**Risk index method**		**Hazard grading method**		**ICMM method**		**Synthesis index method**		**Exposure ratio method**	
				**Risk level**	**RR**	**Risk level**	**RR**	**Risk level**	**RR**	**Risk level**	**RR**	**Risk level**	**RR**
A	Cleaning	13	1.7	Extreme	1	High	1	High	1	Moderate	0.60	High	0.8
	Molding sand	21	1.7	Extreme	1	High	1	High	1	Moderate	0.60	Extreme	1
	Melting	15	1.6	Extreme	1	High	1	High	1	Moderate	0.60	Extreme	1
B	Melting	1	3.33	Extreme	1	High	1	High	1	High	0.80	Extreme	1
	Molding sand	1	1.00	High	0.8	High	1	High	1	High	0.80	Extreme	1
	Casting	1	2.33	Extreme	1	High	1	High	1	High	0.80	Extreme	1
C	Casting	5	15	Extreme	1	High	1	High	1	High	0.80	Extreme	1
D	Cleaning	23	1.667	Extreme	1	High	1	High	1	Moderate	0.60	Extreme	1
	Casting	23	2.333	Extreme	1	High	1	High	1	Moderate	0.60	Extreme	1
E	Grinding	2	4.75	Extreme	1	High	1	High	1	High	0.80	Extreme	1
F	Casting	1	0.8	Moderate	0.6	High	1	Moderate	0.75	High	0.80	Extreme	1
G	Melting	4	0.75	Moderate	0.6	Moderate	0.75	Moderate	0.75	High	0.80	Extreme	1
	Casting	4	3.17	Extreme	1	High	1	High	1	High	0.80	Extreme	1
	Molding sand	8	0.83	High	0.8	High	1	Moderate	0.75	High	0.80	Extreme	1
H	Grinding	6	0.725	High	0.8	Moderate	0.75	Moderate	0.75	High	0.80	Extreme	1
	Casting	5	1.355	Extreme	1	High	1	High	1	High	0.80	Extreme	1
	Molding sand	4	1.406	Extreme	1	High	1	High	1	High	0.80	Extreme	1
	Cleaning	2	1.587	Extreme	1	High	1	High	1	High	0.80	Extreme	1
I	Cleaning	3	2.798	Extreme	1	High	1	High	1	High	0.80	Extreme	1
	Casting	3	2.163	Extreme	1	High	1	High	1	High	0.80	Extreme	1
	Grinding	3	1.908	Extreme	1	High	1	High	1	High	0.80	Extreme	1
	Melting	1	1.153	High	0.8	High	1	High	1	High	0.80	Extreme	1
J	Casting	3	0.768	High	0.8	High	1	Moderate	0.75	High	0.80	High	0.8
	Molding sand	2	0.712	Moderate	0.6	Moderate	0.75	Moderate	0.75	High	0.80	High	0.8
	Cleaning	3	0.81	High	0.8	High	1	Moderate	0.75	High	0.80	High	0.8
K	Cleaning	2	2.16	Extreme	1	High	1	High	1	High	0.80	Extreme	1
	Melting	1	1.99	Extreme	1	High	1	High	1	High	0.80	Extreme	1
	Casting	2	2.04	Extreme	1	High	1	High	1	High	0.80	Extreme	1
	Molding sand	1	2.08	Extreme	1	High	1	High	1	High	0.80	Extreme	1
L	Cleaning	2	1.9	Extreme	1	High	1	High	1	High	0.80	Extreme	1
	Molding sand	2	1.87	Extreme	1	High	1	High	1	High	0.80	Extreme	1
	Melting	1	0.58	Moderate	0.6	Low	0.5	Moderate	0.75	High	0.80	High	0.8
	Casting	3	0.87	High	0.8	High	1	Moderate	0.75	High	0.80	Extreme	1
M	Melting	1	1.2	High	0.8	High	1	High	1	High	0.80	Extreme	1
	Casting	1	1.54	Extreme	1	High	1	High	1	High	0.80	Extreme	1
	Molding sand	1	1.75	Extreme	1	High	1	High	1	High	0.80	Extreme	1
	Cleaning	1	4.5	Extreme	1	High	1	High	1	High	0.80	Extreme	1
N	Grinding	1	1.5	Extreme	1	High	1	High	1	High	0.80	Extreme	1
	Casting	2	1.75	Extreme	1	High	1	High	1	High	0.80	Extreme	1
	Molding sand	1	1.66	Extreme	1	High	1	High	1	High	0.80	Extreme	1
	Cleaning	2	2.00	Extreme	1	High	1	High	1	High	0.80	Extreme	1
O	Casting	1	1.4	Extreme	1	High	1	High	1	High	0.80	Extreme	1
	Melting	1	1.2	High	0.8	High	1	High	1	High	0.80	Extreme	1
	Molding sand	2	1.4	Extreme	1	High	1	High	1	High	0.80	Extreme	1
	Cleaning	1	3.3	Extreme	1	High	1	High	1	High	0.80	Extreme	1
P	Cleaning	2	0.873	High	0.8	High	1	Moderate	0.75	High	0.80	Extreme	1
	Casting	1	0.731	Moderate	0.6	High	1	Moderate	0.75	High	0.80	Extreme	1
	Molding sand	3	0.9	High	0.8	High	1	High	1	High	0.80	Extreme	1
Q	Cleaning	3	1.55	Extreme	1	High	1	High	1	High	0.80	Extreme	1
	Melting	3	0.652	Moderate	0.6	Moderate	0.75	Moderate	0.75	High	0.80	High	0.8
	Casting	3	1.162	High	0.8	High	1	High	1	High	0.80	High	0.8
	Molding sand	6	0.765	High	0.8	Moderate	0.75	Moderate	0.75	High	0.80	High	0.8
R	Casting	2	1.50	Extreme	1	High	1	High	1	High	0.80	Extreme	1
	Grinding	1	1.37	Extreme	1	High	1	High	1	High	0.80	Extreme	1
	Cleaning	1	1.54	Extreme	1	High	1	High	1	High	0.80	Extreme	1
S	Molding sand	8	2.297	Extreme	1	High	1	High	1	High	0.80	Extreme	1
	Cleaning	2	6.713	Extreme	1	High	1	High	1	High	0.80	Extreme	1
	Grinding	2	1.753	Extreme	1	High	1	High	1	High	0.80	Extreme	1
T	Casting	5	2.8	Extreme	1	High	1	High	1	Moderate	0.60	Extreme	1
	Grinding	5	0.4	Mild	0.4	Mild	0.5	Moderate	0.75	Moderate	0.60	High	0.8
U	Casting	3	0.6	Moderate	0.6	Moderate	0.75	Moderate	0.75	Moderate	0.60	High	0.8
	Grinding	4	1.5	Extreme	1	High	1	High	1	Moderate	0.60	Extreme	1
V	Molding sand	6	2.0	Extreme	1	High	1	High	1	High	0.80	Extreme	1
W	Molding sand	4	1.3	Extreme	1	High	1	High	1	High	0.80	Extreme	1
X	Molding sand	2	1.3	Extreme	1	High	1	High	1	High	0.80	Extreme	1
Y	Grinding	1	1.0	High	0.8	High	1	High	1	High	0.80	Extreme	1
	Cleaning	2	1.0	High	0.8	High	1	High	1	High	0.80	Extreme	1

### Correlation analysis among evaluation results using five methods

The Index method showed strong correlations with the hazard grading method and the ICMM method (*r* = 0.609 and 0.798, respectively), and moderate correlation with the exposure ratio method (*r* = 0.541) (*P* < 0.001). The hazard grading method exhibited strong correlations with the ICMM method (*r* = 0.685) and the exposure ratio method (*r* = 0.628) assessment results (*P* < 0.001). There were no statistically significant differences in Spearman correlation coefficients between the synthesis index method and the other four methods (*P* > 0.05). The specific R values are listed in Table 2.

**Table 2:** Spearman correlation analysis between risk ratios of different assessment models.

	**RR_Riskindexmethod_**	**RR_Hazardgradingmethod_**	**RR_ICMM_ _method_**	**RR_synthesisindexmethod_**	**RR_exposureratiomethod_**
RR_Riskindexmethod_	1				
RR_Hazardgradingmethod_	0.609^a^	1			
RR_ICMM_	0.798^a^	0.685^a^	1		
RR_synthesisindexmethod_	−0.050	0.133	−0.002	1	
RR_exposureratiomethod_	0.541^a^	0.628^a^	0.579^a^	0.203	1

^a^*P* < 0.05 by Spearman correlation analysis.

### Results of Kappa consistency test

The risk index method exhibited poor agreement with the hazard grading method, ICMM method, and exposure ratio method, with Kappa values of 0.251 (*P* = 0.002), 0.300 (*P* < 0.001), and 0.268 (*P* = 0.003), respectively. The hazard grading method showed moderate agreement with the ICMM method and exposure ratio method, with Kappa values of 0.547 (*P* < 0.001) and 0.497 (*P* < 0.001), respectively. The ICMM method demonstrated moderate agreement with the exposure ratio method, with a Kappa value of 0.561 (*P* < 0.001). The synthesis index method displayed poor agreement with the other four evaluation methods (*P* > 0.05). The specific Kappa values are listed in Table 3.

**Table 3:** Consistency analysis between risk ratio levels of different assessment models.

	**RR_Riskindexmethod_**	**RR_Hazardgradingmethod_**	**RR_ICMM_ _method_**	**RR_synthesisindexmethod_**	**RR_exposureratiomethod_**
RR_Riskindexmethod_	1				
RR_Hazardgradingmethod_	0.251^a^	1			
RR_ICMMmethod_	0.300^a^	0.547^a^	1		
RR_synthesisindexmethod_	0.039	0.009	< 0.001	1	
RR_exposureratiomethod_	0.268^a^	0.497^a^	0.561^a^	−0.028	1

Kapa consistency analysis showed.

^a^*P* < 0.05.

## Discussion

According to *Henan Issued The Foundry Discipline And Foundry Industry Development Report* (21) indicates that since the year 2000, China's total output of castings has maintained its position as the world's top producer for 15 consecutive years. Among China's casting industry, Henan stands out as a leading province, rightfully earning the title of a casting powerhouse. Ferrous metal casting industry is an important branch of the foundry industry, which mainly refers to the process of producing cast ferrous metal (e.g., cast iron, cast steel, etc.) parts and products. Silica dust as a major occupational disease hazard factor in the ferrous metal foundry industry (22), and the long-term exposure of workers to the working environment containing silica dust may lead to occupational diseases, such as silicosis, etc. (23). It is necessary to assess the risk level of silica dust in the ferrous metal foundry industry and find the most suitable risk assessment method to provide a basis for the application of risk assessment techniques in the key industries of silica dust occupational exposure.

Previous studies have shown that current OHRA methods have different scopes of application and limitations (3, 24, 25). In this study, we employed five methods—the risk index method (14), the hazard grading method (8), the ICMM method (9), the synthesis Index method (10), and the exposure ratio method (10)—to assess the occupational health risks of job positions in 25 black metal casting enterprises where silica dust concentrations exceeded occupational exposure limits. The assessment results of the five methods were not identical, and the overall consistency was poor, which may be due to the fact that the use of the synthesis index method for the assessment incorporated multiple factors and considered the protection situation, resulting in a decrease in the weighting of E/OEL, which was in line with the existing literature (26). The other four assessment methods incorporate fewer factors and produce unstable results when the concentration of hazardous factors is too high or too low. For example, in the assessment of jobs with silica dust concentrations exceeding twice the occupational exposure limit in this survey, the risk index method calculates that if the concentration of occupational disease hazards increases, the risk index rises by an exponential multiple of 2, and this change largely affects the final assessment results; The hazard grading method stipulates that when B > 2, WB is taken as WB = B, making it easy for occupational disease hazards with concentrations exceeding twice the occupational exposure limit to be classified as highly hazardous. The ICMM method primarily evaluates based on worker exposure levels and the classification of adverse health consequences, which are largely influenced by the subjective judgment of the researchers. The results may exhibit subjective bias, leading to high-risk assessment results when the concentration of occupational disease hazards is significantly higher than the exposure limit, consistent with existing research findings (27, 28).

Similarly, when assessing jobs where silica dust concentrations do not exceed twice the occupational exposure limit, the results are not exactly the same. Taking the grinding post of enterprise T as an example, the silica dust concentration of this post is 0.4 mg/m^3^, and the exposure ratio is 1.3, which is far lower than the average of the exposure ratio of 67 posts of 6.31. When applying the exponential method of assessment, the 1.3th power of 2 does not similarly, the exposure ratio of 1.3 in the hazard classification method corresponds to a weighting of WB of 1, which makes the risk level of the assessment result a mild hazard. When applying the ICMM matrix method of assessment, too few factors are considered in the matrix design and the model categories are roughly delineated, which may lead to an unrealistic distribution of risk levels (29), and the assessor tends to think that the potential health consequences are not serious at this exposure level, which makes it easy to underestimate the risk. Both the synthesis index method and the exposure ratio method determined that the exposure level was high and the exposure time was long, but the synthesis index method included many calculation factors and took into account that the post was well-protected and had good protective effects, which resulted in a medium-risk result, while the exposure ratio method did not take into account the effect of protective measures on the actual exposure concentration, and the post was judged to be high-risk. The inconsistency of such assessment results not only affects the safety management decisions of enterprises, but may also have a long-term negative impact on the health of workers, while at the same time leading to public skepticism about the responsibility of enterprises and damaging their reputation. Therefore, the establishment of a more scientific and systematic OHRA mechanism and the strengthening of monitoring and control of occupationally hazardous factors are not only necessary measures to protect workers' health, but also key to promoting the sustainable development of enterprises.

The above results show that although each OHRA method has its own characteristics and limitations, they can complement each other in practical application, thus providing multi-faceted support for occupational hazard assessment. Therefore, in risk assessment of enterprises with excessive occupational hazards but effective protective measures, we should collect as much detailed information as possible on exposure concentration, vapor pressure, usage, exposure time, protective effect of protective measures, etc., and then use the synthesis index method in combination with other assessment methods to conduct the assessment, which can to some extent make up for the shortcomings of other assessment methods that are prone to overestimation of the risk level under the conditions.

In summary, this study emphasizes the diversified methodological choices in occupational risk assessment and points out the applicability of each method to silica dust-exposed jobs in the ferrous metal foundry industry. Future studies can further explore the applicability of different methods in specific industries and work environments, and thus develop more accurate occupational hazard assessment models. Meanwhile, the combination of modern technological tools, such as big data analysis and machine learning, can enhance the automation and accuracy of the assessment process and provide more scientific decision support for the management of occupational safety and health.

## Data Availability

The raw data supporting the conclusions of this article will be made available by the authors, without undue reservation.
